# Re-analyzing the SARS-CoV-2 series using an extended integer-valued time series models: A situational assessment of the COVID-19 in Mauritius

**DOI:** 10.1371/journal.pone.0263515

**Published:** 2022-02-08

**Authors:** Ashwinee Devi Soobhug, Homeswaree Jowaheer, Naushad Mamode Khan, Neeshti Reetoo, Kursheed Meethoo-Badulla, Laurent Musango, Célestin C. Kokonendji, Azmi Chutoo, Nawel Aries

**Affiliations:** 1 Statistics Mauritius, Ministry of Finance, Economic Planning and Development, Port Louis, Mauritius; 2 Department of Economics and Statistics, University of Mauritius, Moka, Mauritius; 3 Department of Health And Wellness, Ministry of Education, Tertiary Education, Science and Technology, Vacoas-Phoenix, Mauritius; 4 Communicable Disease Control Unit, Ministry of Health and Wellness, Port Louis, Mauritius; 5 World Health Organization Country Representative in Mauritius, Port Louis, Mauritius; 6 Laboratoire de Mathématiques de Besançon, UMR 6623 CNRS-UBFC, Université Bourgogne Franche-Comté, Besançon, France; 7 Department of Mathematics, University of Bangui, Bangui, Central African Republic; 8 Faculty of Mathematics, University of Science and Technology Houari Boumediene, Algiers, Bab Ezzouar, Algeria; Nantong University, CHINA

## Abstract

This paper proposes some high-ordered integer-valued auto-regressive time series process of order *p* (INAR(*p*)) with Zero-Inflated and Poisson-mixtures innovation distributions, wherein the predictor functions in these mentioned distributions allow for covariate specification, in particular, time-dependent covariates. The proposed time series structures are tested suitable to model the SARs-CoV-2 series in Mauritius which demonstrates excess zeros and hence significant over-dispersion with non-stationary trend. In addition, the INAR models allow the assessment of possible causes of COVID-19 in Mauritius. The results illustrate that the event of Vaccination and COVID-19 Stringency index are the most influential factors that can reduce the locally acquired COVID-19 cases and ultimately, the associated death cases. Moreover, the INAR(7) with Zero-inflated Negative Binomial innovations provides the best fitting and reliable Root Mean Square Errors, based on some short term forecasts. Undeniably, these information will hugely be useful to Mauritian authorities for implementation of comprehensive policies.

## Introduction

In early March 2021, Mauritius was struck by a second wave of the Novel Coronavirus 2019 (COVID-19) pandemic among the local community after officially recording a long sequence of zero locally acquired active cases. In fact, it is worth to mention that during the first wave of the COVID-19 pandemic, and especially after detecting the first set of local cases, on 18 March 2020, Mauritius implemented timely strict sanitary measures in terms of national lockdown, safe shopping guidelines, mandatory face covering in public places, minimal public gathering followed by COVID-19 related legislations like the Quarantine Bill and COVID-19 Miscellaneous Bill [1]. Moreover, as a pro-active strategy, the vaccination campaigns among front-liners in the disciplined forces and health sectors, kick-started in January 2021. A variety of vaccines notably Oxford-AstraZeneca/Covishield, Covaxin and Sinopharm were obtained from country partners and consequently, the targeted audience for vaccination expanded, covering old-aged persons, people with comorbidities, and personnel working in the education, retail and other economic sectors. As at May 2021, around 18 percent of the total Mauritian population has already received the first jab of the vaccine. This process is still ongoing with aim to vaccinate at least 60% of the population in general, by end of July 2021 thus attaining herd-immunity before opening of the frontier. Even the vaccination exercise for the second doses has already started and has been running successfully.

The second wave which was of a sporadic transmission mode based on some identifiable clusters, was immediately controlled by the authorities. The Ministry of Health and Wellness accelerated the contact tracing exercise and to contain the virus more rapidly, law enforcers implemented novel localised mobility restrictions in regions (“red-zoned” areas) with large number of contaminations under the Temporary Restrictions of Movement Order. In terms of evidenced based policies, Mauritius is indeed well positioned but on the other hand, the uncommon patterns in the COVID-19 series raise some concerns in the research community especially in the midst of statistics and data analytics. The COVID-19 new cases series in Mauritius has some distinctive features like a purely unique serial trend with excess of zeros and some oscillations, leading to over-dispersion, while the corresponding COVID-19-related death series describes a preponderance of excess zeros. These series thus imply that the simple integer-valued auto-regressive model (INAR(1)) with Poisson or extra-Poisson innovations is surely insufficient in this context [2–4] and ignoring the excess of zeros will lead to biasedness in the estimated parameters and standard errors [5]. To remedy, this paper proposes to construct a novel high-ordered integer-valued auto-regressive process (INAR) with Zero-Inflated (ZI) innovation distributions. This novel construction bridges two important gaps. Firstly, as seen in the literature, the ZI models have extensively been used in regression contexts only (See [6, 7] and the references therein) while its applications in counting time series modelling is quite restricted to first order only [8–17].

Secondly, the proposed time series model allows for covariate specification, which in the context of the COVID-19 analysis, is primordial. In fact, it is important to identify the significant factors contributing to the propagation of SARS-CoV-2 in the local community, while also detecting the expected impact of the covariates on the COVID-19 infection in the local community. Thereon, such information will extremely be useful to the local concerned authorities and for forecasting purposes. As regards to the factors, in the first wave, several factors such as public health measures, strong political engagement, stricter legislations, population behaviour to established sanitary norms [18], sensitization campaigns and the institution of quarantine centers were found to successfully curb the spread of the virus [1]. Considering the second wave, new covariates like the reproduction rate (ReR), the COVID-19 Risk due to weather conditions (CRW), the major event of vaccination and the COVID-19 Stringency Index need to be assessed in the Mauritian context. Besides, in the European and Asian regions, ReR [19–21], CRW [22, 23] and vaccinations [24] have largely demonstrated their association with COVID-19 transmission while during the first wave in Mauritius, the COVID-19 Stringency index was the most significant factor in curbing the virus [4]. Further details on the covariates are provided in Section 3. An accurate forecasting with acceptable RMSE is also targeted because most restorative and preparedness policy decisions, be it in terms of adequate vaccines, financial requirement and re-opening of frontier, will be based on the new COVID-19 cases’ projections.

The organization of the paper is as follows: In Section 2, the local active COVID-19 data structures and its descriptive statistics are provided. Section 3 emphasizes on the INAR model construction with some novel innovations and ZI distributions. The inferential properties of the INAR process are also discussed. Section 4 focuses on the fitting of the various INAR models and providing the possible short term forecasts. This section also comprises of the discussions on the several significant factors. The concluding remarks and some limitations are provided in Section 5.

## Materials and methods

### The SARS-CoV-2 series data for Mauritius

**Fig 1:**
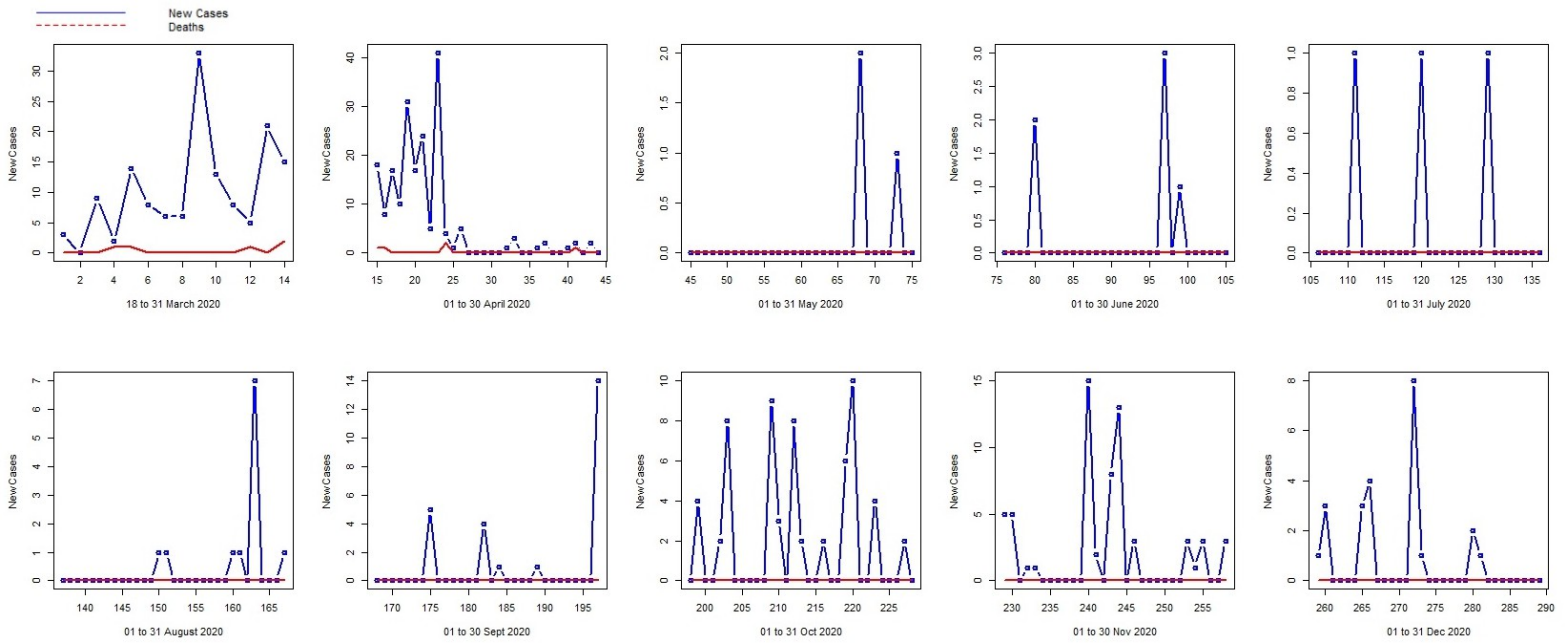
The COVID-19 cases versus death series in 2020.

The daily new COVID-19 infection and death series for Mauritius, covering the period from 18 March 2020 to 25 April 2021, summing to a total of 404 observations, were extracted from the official portal for European data (See https://data.europa.eu/data/datasets/covid-19-coronavirus-data?locale=en). The evolution of the COVID-19 new infection cases series and the death series are displayed below:

From Fig 1, it can be deduced that at the beginning of COVID-19 pandemic, the situation was worrisome with the climbing number of daily deaths cases(in red), associated with the increasing trend in the number of new daily COVID-19 infection cases (in blue). As from April 2020 till December 2020, the spread of the SARS-CoV-2 in the local community has plunged and a long sequence of zero daily locally acquired new COVID-19 cases and deaths cases were reported. Note that a few COVID-19-related death cases were reported in March and April 2020, especially among patients with comorbidities. Next, new imported cases of COVID-19 infection cases were detected in October 2020 after the frontier was re-opened but these were successfully mitigated in quarantine centers. The mandatory 14-days of isolation in established quarantine centers proved its optimal effectiveness.

**Fig 2:**
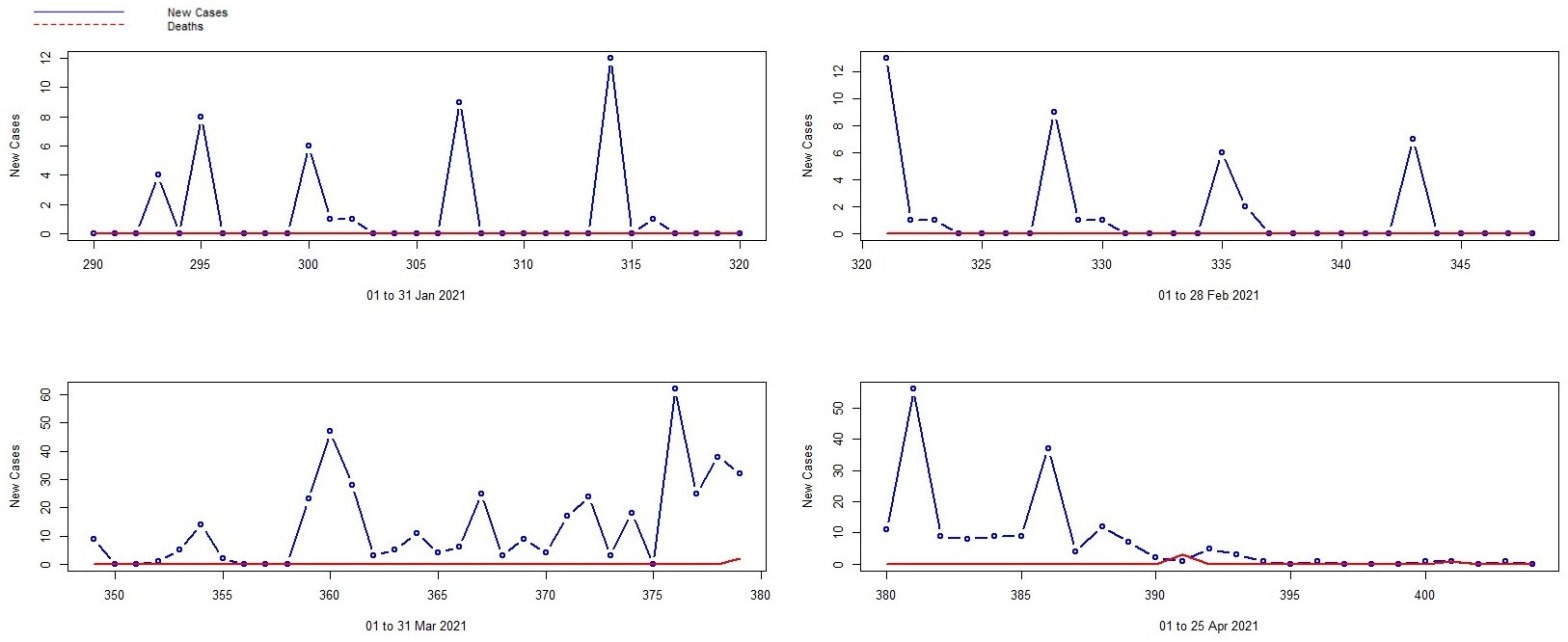
The COVID-19 cases versus deaths series in 2021.

Fig 2 covers the period from January to April 2021. In January and February 2021, a few active local cases among front-liners were reported but by accelerating the vaccination campaigns, the severity of the disease was reduced. It was in March 2021, that a sudden increase in the number of local infection was reported among some identifiable clusters. As for the death series, a long series of zero cases were reported.

**Fig 3:**
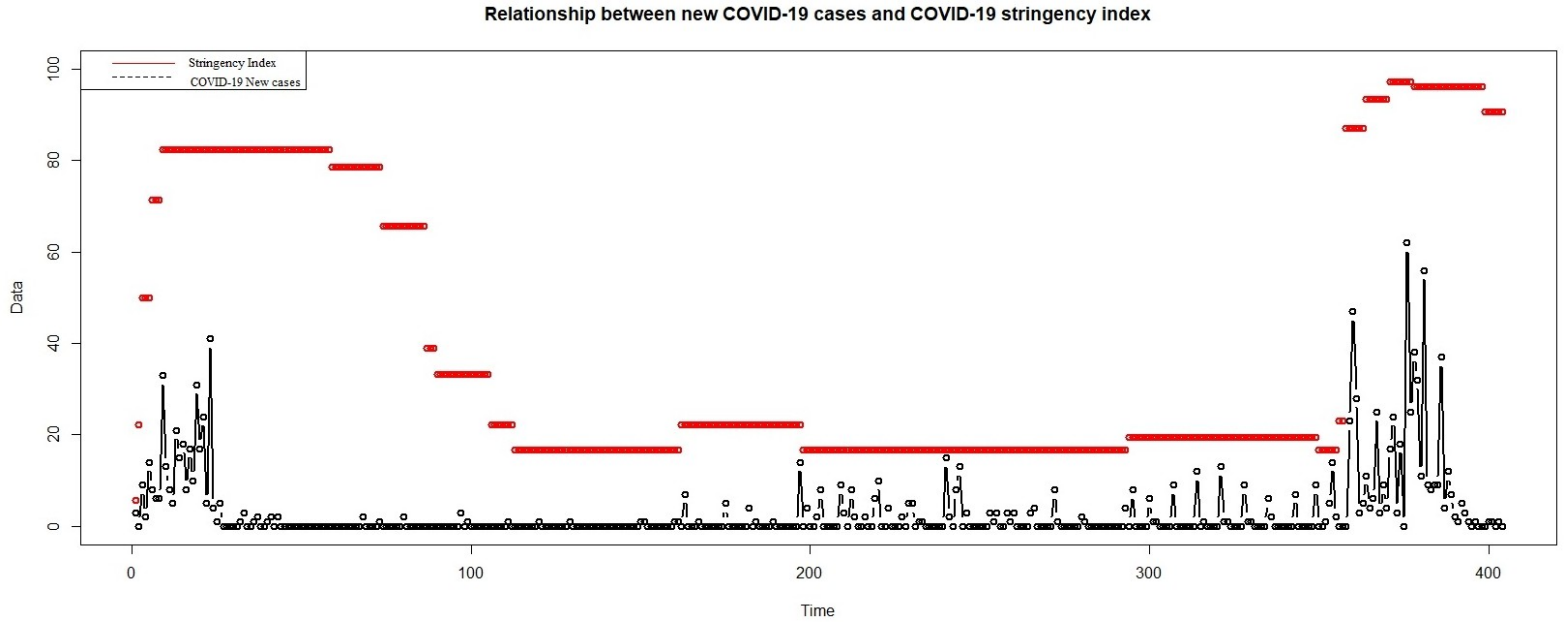
The relationship between new COVID-19 cases and COVID-19 stringency index.

The relationship between the COVID-19 Stringency index in Mauritius (Refer to https://ourworldindata.org/covid-stringency-index) and the number of daily new locally acquired COVID-19 active cases also plays a significant role, as proven in [4] and as shown in Fig 3 above. The COVID-19 Stringency index was near 100 in March 2020 considering the immediate imposition of various sanitary measures like the sanitary curfew and national lockdown, closure of borders, minimal gathering and other health-related measures. This index moved from above 80 to below 25 from July 2020 to February 2021 because since the number of new COVID-19 cases were decreasing considerably, the sanitary restrictions were lessened. However, post the resurgence in March 2021, with the re-introduction of the sanitary measures, the index again shot upward to 100, indicating strictness of the policies.

In Table 1 below, we provide the descriptive statistics and preliminary test results to confirm the nature of both data series along with their respective Auto Correlation Function (ACF) and Partial Auto Correlation Function (PACF) plots to determine the orders:

**Table 1:** Descriptive statistics and test results for COVID-19 active cases and deaths series in Mauritius.

Descriptive Statistics	New COVID-19 cases	Deaths
Mean	3.0	0.04
Variance	57.9	0.07
Vuong and Jan Van den Broek tests for zero-inflation	2e-16	2e-16
Over-dispersion test using qcc	0	0
Cox-Stuart test for presence of trend	4.82e-05	0.066
Box-Ljung	2e-16	0.01
Order	7	7

Based on the *qcc.overdispersion.test* in R statistical software via the ‘*qcc*’ package, it is confirmed that the data is over-dispersed and due to the excess zeros in the data, the Vuong test, refer to Table 5 in S1 Appendix, and Van den Broek tests were also significant, proving that the series is zero-inflated as well. The Ljung-Box test ascertains the existence of serial correlation in the series. The presence of trend was also significant. In fact, via the Cox-Stuart tests, it was found that the COVID-19 new cases has a decreasing trend and death series does not have an increasing trend. Details on these tests are shown in the S1 Appendix.

**Fig 4:**
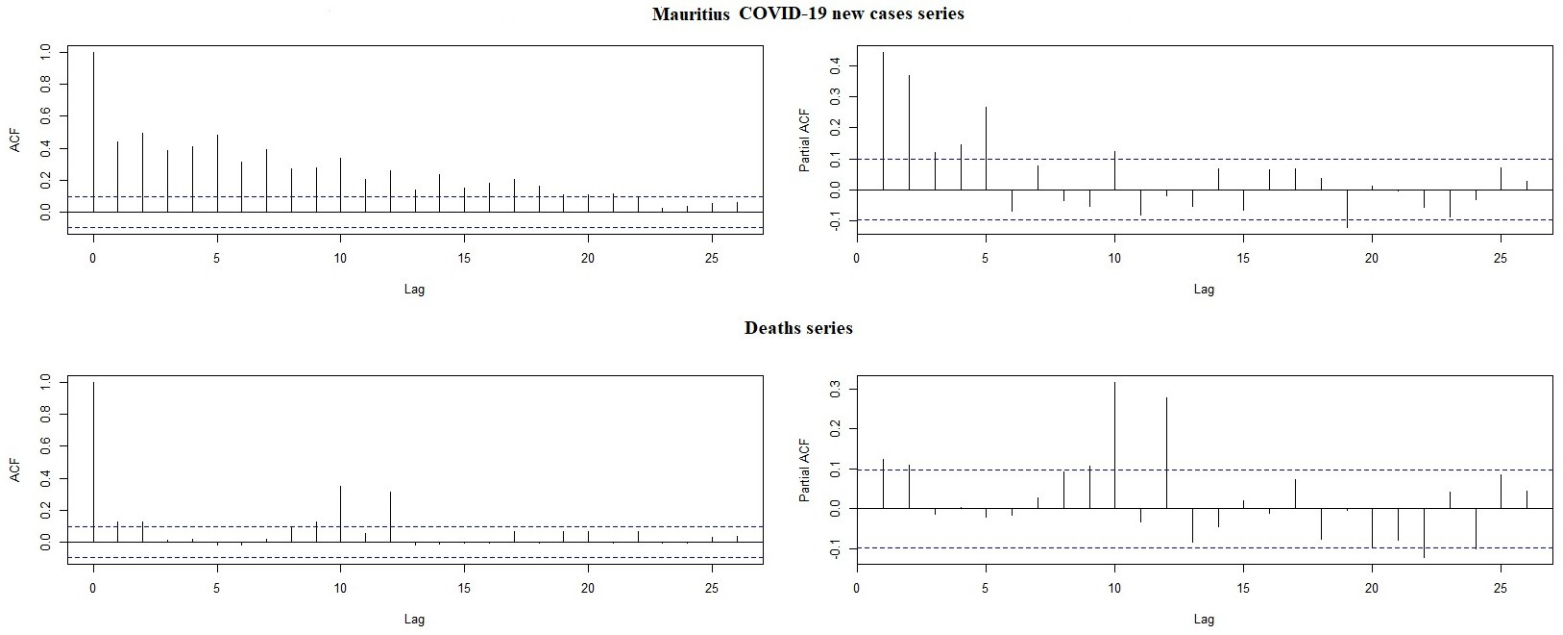
The ACF and PACF plots for COVID-19 new cases and death series.

Refer to Fig 4, the ACF plots for both series demonstrate a slow decaying over 20 lags, and this is a basis of non-stationary for the time-series while the PACF plots confirm that both series are high-ordered (order = 7).

To address the non-stationarity issue, it is thus proposed to allow for covariate specification, which in the context of the COVID-19 analysis, is important. The following time-dependent covariates were considered:

- The COVID-19 Stringency Index (SI): This variable has been calculated from nine metrics, namely school closures; workplace closures; cancellation of public events; restrictions on public gatherings; closures of public transport; stay-at-home requirements; public information campaigns; restrictions on internal movements; and international travel controls and is available on a daily basis at https://ourworldindata.org/covid-stringency-index. This index gives an indication of the strictness of government policies to the COVID-19 pandemic. The score is between 0 to 100 where an index nearing 100 indicates strict response otherwise less strict response [25]. In this study, the logarithm of the nominal value of the SI (log(SI)) was used for analysis purposes.
- The event of vaccination (Vaccine): This is an important time-varying variable because as at date, the vaccine roll-outs in Mauritius is rising given the authorities’s aim to achieve herd immunity. More than 1 million vaccine doses have already been obtained through bilateral agreements with India and China even though there is an intense competition between countries for the purchase the COVID-19 vaccine. For this study, the event of vaccination was categorised into two possibilities (binary) where 1 indicates that the event for vaccination is being done and 0 (ref) that the event of vaccination is not being done. Data on vaccination was obtained from https://ourworldindata.org/covid-vaccinations [26] and from the official COVID-19 platform in Mauritius, falling under the aegis of the Ministry of Health and Wellness https://covid19.mu/.
- The reproduction rate (ReR): This covariate refers to the degree of propagation of SARS-CoV-2 from one person to another. The data on ReR was obtained from https://ourworldindata.org/covid-cases and the logarithm of the nominal value of ReR (log(ReR)) was considered. To note that this reproduction rate relates to the degree of transmissibility of the “original” SARS-CoV-2 and was used as a proxy to understand the severity of the coronavirus, considering that nowadays, constant mutation of the SARS-CoV-2 has been observed. In terms of proactive health related measures, this rate can be highly indicative. The logarithm of the nominal value of ReR (log(ReR)) was considered.
- The Relative COVID-19 Risk due to Weather and Air Pollution (CRW): This variable represents some environmental factors and explains their impact on COVID-19 transmission. Weather factors like average and diurnal temperature, ultraviolet (UV) index, humidity, pressure, precipitation and air pollutants (SO2 and Ozone) were considered while computing this index. In this paper, the CRW was categorised into 0 and 1 (binary) where 0 (ref) is when an index is below 1 referring to relatively lower impact of weather factors on spread of COVID-19 and 1 is when an index is above 1, indicating otherwise. Data has been extracted from https://projects.iq.harvard.edu/covid19 and imputation based on observations were done for missing values.

Also, to cater for the long sequence of zeros and high autocorrelation, this paper bring forward the Zero-inflated (ZI) with different Poisson-mixture innovations models and the INAR(*p*) models because as illustrated in [2–4], ignoring the excess of zeros will lead to biasedness in the estimated parameters and standard errors [5]. More on these novel ZI models and the inferential part of the general INAR processes are provided in the subsequent Section.

### The Zero-inflated poisson mixture models

The Zero Inflated (ZI) models, introduced by [6], are suitable for over-dispersed count data that exhibit excessive zeros. These data are commonly encountered in social sciences, likewise in the analysis of drug addicts [27], crimes [28], adolescents’ drinking patterns [29], counselling session attendance [30], or in the financial sectors such as in the modelling of insurance claims [31], and in health studies such as in dental caries [32], in injection cessation in HIV patients [33] and among many other applications areas mentioned in [7].

Basically the ZI models is a mixture of two distributions: Firstly, a probability distribution that degenerates at zero and on the second stage, mixed with a standard probability model such as the Poisson or Negative Binomial (NB) model. The general form is given by:

$$\begin{array}{c} P\left(R_{t}=r_{t}\right)=\pi_{t}g_{1}\left(R_{t}\right)+\left(1-\pi_{t}\right)g_{2}\left(R_{t}\right) \end{array}$$

where *π*_*t*_, the mixing proportion and lies in the interval between 0 and 1, indicates as well the rate of zero inflation and *g*_1_(.) and *g*_2_(.) are the corresponding densities.

By replacing *g*_1_(.) with a probability distribution that generates at zero and *g*_2_(.) by the Poisson distribution with parameter λ_*t*_, we derive the ZI Poisson (ZIP) as:

$$\begin{array}{c} P\left(R_{t}=r_{t}\right)=\pi_{t}+\left(1-\pi_{t}\right)e^{-\lambda_{t}}, \end{array}$$

$$\begin{array}{c} P\left(R_{t}=r_{t}\right)=\left(1-\pi_{t}\right)\frac{{\lambda_{t}}^{r_{t}}e^{-\lambda_{t}}}{r!},t=1,2,3,\ldots \end{array}$$

and the corresponding probability generating function (PGF) is

$$\begin{array}{c} G_{R_{t}}\left(s\right)=\pi_{t}+\left(1-\pi_{t}\right)e^{\lambda_{t}\left(s-1\right)}. \end{array}$$ (1)

Similarly we can write the ZI Negative Binomial (ZI NB) with parameter (λ_*t*_, *ν*^−1^)

$$\begin{array}{c} P\left(R_{t}=r_{t}\right)=\pi_{t}+\left(1-\pi_{t}\right){\left(\frac{\nu^{-1}}{\lambda_{t}+\nu^{-1}}\right)}^{\nu^{-1}}, \end{array}$$

$$\begin{array}{c} P\left(R_{t}=r_{t}\right)=\left(1-\pi_{t}\right)\{\frac{\Gamma (R_{t}+\nu^{-1})}{r!\Gamma (\nu^{-1})}{\left(\frac{\nu^{-1}}{\lambda_{t}+\nu^{-1}}\right)}^{\nu^{-1}}{\left(\frac{\lambda_{t}}{\lambda_{t}+\nu^{-1}}\right)}^{R_{t}}\},r=1,2,3,\ldots ; \end{array}$$

$$\begin{array}{c} G_{R_{t}}\left(s\right)=\pi_{t}+\left(1-\pi_{t}\right){\left[1+\nu \lambda_{t}\left(1-s\right)\right]}^{-\nu^{-1}}; \end{array}$$

and, recently, [34] proposed the ZI COM-Poisson model (ZI-CMP) where,

$$\begin{array}{c} P\left(R_{t}=r_{t}\right)=\pi_{t}+\left(1-\pi_{t}\right)\frac{1}{Z(\lambda_{t},\nu )}, \end{array}$$

$$\begin{array}{c} P\left(R_{t}=r_{t}\right)=\left(1-\pi_{t}\right)\frac{1}{Z(\lambda_{t},\nu )}\frac{{\lambda_{t}}^{R_{t}}}{{\left(R_{t}!\right)}^{\nu }},t=1,2,3,\ldots \end{array}$$

and its PGF is

$$\begin{array}{c} G_{R_{t}}\left(s\right)=\pi_{t}+\left(1-\pi_{t}\right)\frac{Z(\lambda_{t}s,\nu )}{Z(\lambda_{t},\nu )}, \end{array}$$

where the *Z*(λ_*t*_, *ν*) is computed from [35] as:

$$\begin{array}{c} Z\left(\lambda_{t},\nu \right)=\frac{\text{exp}\{\nu {\lambda_{t}}^{1/\nu }\}}{{\lambda_{t}}^{(\nu -1)/2\nu }{\left(2\pi_{t}\right)}^{(\nu -1)/2}\sqrt{\nu }}(1+c_{1}{\left(\nu {\lambda_{t}}^{1/\nu }\right)}^{-1}+c_{2}{\left(\nu {\lambda_{t}}^{1/\nu }\right)}^{-2}+O\left({\lambda_{t}}^{-3/\lambda_{t}}\right)) \end{array}$$ (2)

as λ → ∞ and where,

$$\begin{array}{c} c_{1}=\frac{\nu^{2}-1}{24} \end{array}$$

$$\begin{array}{c} c_{2}-{c_{1}}^{2}/2=\frac{\nu^{2}-1}{48}. \end{array}$$

Next, the Poisson-Tweedie (PT) model in [36] has also shown its efficacy in handling over-dispersed and to some extent, data with excess zero, as discussed in [37–39]. The PGF of the PT model is given by:

$$\begin{array}{c} G_{R_{t}}\left(s\right)=\{\begin{array}{cc} & \text{exp}\{\frac{b}{a}({\left(1-c\right)}^{a}-{\left(1-cs\right)}^{a})\},a\neq 0;\\ & [\frac{{\left(1-c\right)}^{b}}{{\left(1-cs\right)}^{b}}],a=0. \end{array} \end{array}$$ (3)

and its zero-inflated PGF (ZI-PT) is simply given by $\pi_{t}+\left(1-\pi_{t}\right)G_{R_{t}}\left(s\right)$. Furthermore, the PT function can be re-parameterized in terms of λ_*t*_, *σ*^2^, $D_{t}=\frac{\sigma_{t}^{2}}{\lambda_{t}}$ and *a* where;

$$\begin{array}{c} c_{t}=\frac{D_{t}-1}{D_{t}-a}, \end{array}$$

$$\begin{array}{c} b_{t}=\frac{\lambda_{t}{\left(1-c_{t}\right)}^{1-a}}{c_{t}}=\frac{\lambda_{t}{\left(1-a\right)}^{1-a}}{\left(D_{t}-1\right){\left(D_{t}-a\right)}^{-a}}. \end{array}$$

Note the probability distribution of the PT model cannot be generally written in its explicit form (See [37]) whilst its probability values can be computed recursively as in [36] or using the method of [40], explained in the next subsection. It also important to note that *a* = 0 in Eq (3) corresponds to NB.

Apart from these models, the recently studied Cosine-Geometric models [4, 41] is also proven useful for count data modelling. The PGF of WCG is given by:

$$\begin{array}{c} G_{R_{t}}\left(s\right)=\frac{C_{\lambda_{t}^{*},\nu }}{2}[\frac{1}{1-\lambda_{t}^{*}s}+\frac{1-\lambda_{t}^{*}s\;\text{cos}\left(2\nu \right)}{1-2\lambda_{t}^{*}s\;\text{cos}\left(2\nu \right)+{\left(\lambda_{t}^{*}s\right)}^{2}}],\;s<-\text{ln}\left(\lambda_{t}^{*}\right), \end{array}$$

where $\lambda_{t}^{*}=\frac{\lambda_{t}}{1+\lambda_{t}}$ and $\lambda_{t}^{*}\in (0,1)$ and $\nu \in [0,\frac{\pi }{2})$. The PGF of the ZI-WCG is $\pi_{t}+(1-\pi_{t})\times G_{R_{t}}(s)$.

In the event we have some explanatory variables, given by the vector *x*_*t*_, which are known to influence the *t*^*th*^ response variable *y* = *y*_*t*_, then *x*_*t*_ = [*x*_1_, *x*_2_, …, *x*_*p*_], and $\lambda_{t}=\text{exp}\left({x_{t}}^{T}\beta \right)$, for the *t*^*th*^ term. In this context, *p* = 4, with *x*_1_ = ReR, …, *x*_4_ = CRW. For the zero inflated part, we assume the probability of zero is denoted by *π*_*t*_ for *y* = *y*_*t*_, where $\pi_{t}=\frac{\text{exp}({x_{t}}^{T}\beta )}{1+\text{exp}({x_{t}}^{T}\beta )}$ where *η* = [*η*_1_, *η*_2_, …, *η*_*p*_]^*T*^.

Overall, for the interested reader, the ZI data can easily be generated in R using *ifelse*(*rbinom*(*n*, *size* = 1, *prob* = *π*)>0, 0, *rdis*(*n*, λ*, *ν*)), where for the Poisson model, ‘rdis’ is *rpois(n*,λ = *μ*), for NB model, ‘rdis’ is *rnbinom(n, $\text{size}=\frac{1}{\nu }$, mean=λ)* and for COM-Poisson, ‘rdis’ is *rcmp(n*,λ,*ν*), (similar for the PT model, refer to the *poistweedie* package in R) or alternatively, the data can be obtained from ZIM [42], iZID [43], bZinb [44] and ZiC packages [45].

Note, since the marginal distribution of the counting series is not known, we follow the approach in [46] by conditioning on $F_{t}=\left[Y_{t-1},Y_{t-2},\ldots ,Y_{t-p}\right]$ to obtain

$$\begin{array}{c} G_{R_{t}|F_{t}}\left(s\right)=[\prod_{l=1}^{p}{\left(1-\rho_{l}+\rho_{l}s\right)}^{Y_{t-l}}]\times G_{R_{t}}\left(s\right) \end{array}$$ (4)

and from here, the probability density values for *Y*_*t*_, *t* = *p* + 1, …, *T* can be obtained using the inversion technique [40].

### INAR (p) model

From [2, 3, 47], the simple integer-valued autoregressive process of order 1 (INAR(1)) is written as:

$$\begin{array}{c} Y_{t}=\rho \circ Y_{t-1}+R_{t}, \end{array}$$ (5)

where *t* = 1, 2, 3, …, *T*, subject to ${\left\{R_{t}\right\}}_{t=1}^{T}$ is an identically and independently distributed set of innovation term with, say, E(*R*_*t*_) = *μ*_*t*_ and $\text{Var}(R_{t})=\sigma_{t}^{2}$. The relation between *R*_*t*_ and the previous lagged observations, *y*_*t*−*k*_, for $k\in Z^{+}$, is given as *Cov*(*Y*_*t*−*k*_, *R*_*t*_) = 0. The operator ‘∘’ is the binomial thinning with constant parameter *ρ*; *ρ* ∈ [0, 1), that is defined from [48–54] as $\rho \circ Y=\sum_{s=0}^{Y}b_{s}\left(\rho \right)$ where *b*_*s*_(*ρ*) is an i.i.d Bernoulli r.v with probabilities *ρ*, and hence *ρ* ∘ *Y*|Y ∼ Binomial(Y,*ρ*), with probability generating function *G*_*ρ*∘*Y*|*Y*_(*s*) = (1 − *ρ* + *ρs*)^*Y*^ and hence,

$$\begin{array}{c} G_{Y_{t}|Y_{t-1}}\left(s\right)={\left(1-\rho +\rho s\right)}^{Y_{t-1}}\times G_{R_{t}}\left(s\right) \end{array}$$

The INAR(*p*) process, based on Eq (1) is extended to:

$$\begin{array}{c} Y_{t}=\rho_{1}\circ Y_{t-1}+\rho_{2}\circ Y_{t-2}+\cdots +\rho_{p}\circ Y_{t-p}+R_{t}, \end{array}$$ (6)

where the Bernoulli sequence {*b*_*j*,*k*_} in *ρ*_*k*_ ∘ *Y*_*t*−*k*_ and {*b*_*j*,*k*′_} in *ρ*_*k*′_ ∘ *Y*_*t*−*k*′_ are independent. Suppose, $F_{t}=\{Y_{t-1},\ldots ,Y_{t-p}\}$, then,

1.
$$\begin{array}{c} f_{y_{t}|F_{t}}=F_{y_{t}|F_{t}}\left(y_{t}\right)-F_{y_{t}|F_{t}}\left(y_{t-1}\right) \end{array}$$
2. From [40],
  $$\begin{array}{c} F_{Y_{t}|F_{t}}=\frac{1}{2}-\frac{\pi }{2}\int_{-\pi }^{\pi }Re[\frac{G_{Y_{t}|F_{t}}\text{exp}\left(-i\alpha \right)\times \text{exp}\left(-i\alpha \right)Y_{t}}{1-\text{exp}(-i\alpha )}]d\alpha \end{array}$$

and hence the log-likelihood equation is obtained as:

$$\begin{array}{cc} \text{Pr}(Y_{t}|Y_{t-1},Y_{t-2},Y_{t-3},\ldots ,Y_{t-p}) & =\sum_{i_{1}=0}^{\text{min}(Y_{t-1},Y_{t})}(\begin{array}{c} Y_{t-1}\\ i_{1} \end{array})\rho_{1}^{i_{1}}{\left(1-\rho_{1}\right)}^{Y_{t-1}-i_{1}}\\ & \times \sum_{i_{2}=0}^{\text{min}(Y_{t-2},Y_{t-i_{1}})}(\begin{array}{c} Y_{t-2}\\ i_{2} \end{array})\rho_{2}^{i_{2}}{\left(1-\rho_{2}\right)}^{Y_{t-2}-i_{2}}\times \cdots \\ & \times \sum_{i_{p}=0}^{\text{min}(Y_{t-p},Y_{t-(i_{1}+i_{2}+\cdots +i_{p})})}(\begin{array}{c} Y_{t-p}\\ i_{p} \end{array})\rho_{p}^{i_{p}}{\left(1-\rho_{p}\right)}^{Y_{t-p}-i_{p}}\\ & \times f_{\epsilon_{t}}\left(Y_{t}-\left(i_{1}+i_{2}+\cdots +i_{p}\right)\right) \end{array}$$ (7)

which is solved using the *optim* function in R. From [51], the vector of unknown parameters denoted by $\hat{\theta }-\theta ∼N\left(0,I\left(\hat{\theta }\right)\right)$ where *I*(*θ*) is the Hessian and is obtained from the *optim*$*hessian* in R.

The properties of the INAR(p) process have been further studied in [4, 46, 55, 56]. The R codes can be made available on request.

## Results

Following the model descriptions and properties, we apply the high ordered INAR to analyze the new COVID-19 infected series. We present the results in the Tables 2 and 3 below:

**Table 2:** Estimates, corresponding standard errors in parentheses and p-values.

Innovation	$\hat{\rho_{1}}$	$\hat{\rho_{2}}$	$\hat{\rho_{3}}$	$\hat{\rho_{4}}$	$\hat{\rho_{5}}$	$\hat{\rho_{6}}$	$\hat{\rho_{7}}$	Intercept	ReR	SI	Vaccine	CRW	$\hat{\eta_{0}}$	$\hat{\eta_{1}}$	$\hat{\eta_{2}}$	$\hat{\eta_{3}}$	$\hat{\eta_{4}}$	AIC	Log-Likelihood
ZI-NB	0.188	0.114	0.111	0.056	0.042	0.041	0.041	0.095	0.063	-0.098	-0.107	0.034	0.028	0.170	-0.369	0.149	0.232	2348.8	2314.8
	(0.000)	(0.000)	(0.000)	(0.000)	(0.000)	(0.000)	(0.000)	(0.001)	(0.000)	(0.000)	(0.001)	(0.128)	(0.001)	(0.000)	(0.000)	(0.001)	(0.125)		
	0.000	0.000	0.000	0.000	0.000	0.000	0.000	0.000	0.000	0.000	0.000	0.788	0.000	0.000	0.000	0.000	0.063		
ZI-Poisson	0.204	0.107	0.001	0.056	0.042	0.041	0.031	0.397	0.092	-0.404	-0.648	0.253	0.168	0.038	0.303	0.056	0.517	9081.5	9047.5
	(0.000)	(0.000)	(0.001)	(0.002)	(0.005)	(0.004)	(0.010)	(0.001)	(0.000)	(0.000)	(0.000)	(0.001)	(0.001)	(0.000)	(0.000)	(0.009)	(0.001)		
	0.000	0.000	0.000	0.000	0.000	0.000	0.001	0.000	0.000	0.000	0.000	0.000	0.000	0.000	0.000	0.000	0.000		
ZI-CMP	0.040	0.009	0.044	0.027	0.047	0.023	0.062	0.705	0.462	-0.399	-0.196	0.162	-0.362	2.645	-1.536	2.045	2.216	7688.6	7654.6
	(0.001)	(0.000)	(0.376)	(0.565)	(0.001)	(0.565)	(0.376)	(0.144)	(0.000)	(0.001)	(0.044)	(0.267)	(5.735)	(1.768)	(0.000)	(5.793)	(4.982)		
	0.000	0.000	0.907	0.962	0.000	0.967	0.869	0.000	0.000	0.000	0.000	0.545	0.950	0.135	0.000	0.724	0.656		
ZI-PT	0.017	0.013	0.013	0.055	0.039	0.039	0.039	0.040	0.019	-0.062	-0.022	0.075	-0.070	0.075	-0.024	-0.122	0.011	8987.1	8953.1
	-(0.004)	-(0.007)	-(0.017)	(0.000)	-(0.001)	-(0.006)	-(0.007)	-(0.065)	(0.000)	-(0.001)	-(0.007)	-(0.056)	-(0.285)	-(0.004)	-(0.027)	-(0.285)	-(0.715)		
	0.000	0.051	0.434	0.000	0.000	0.000	0.000	0.539	0.000	0.000	0.000	0.185	0.805	0.000	0.373	0.668	0.988		
ZI-WCG	0.190	0.099	0.099	0.050	0.045	0.040	0.029	0.199	0.157	-0.265	-0.380	0.171	-0.069	0.240	0.197	0.344	0.074	8344.6	8310.6
	(0.001)	(0.001)	(0.044)	(0.000)	(0.000)	(0.001)	(0.044)	(0.015)	(0.000)	(0.000)	(0.015)	(0.067)	(0.067)	(0.000)	(0.001)	(0.067)	(0.260)		
	0.000	0.000	0.024	0.000	0.000	0.000	0.505	0.000	0.000	0.000	0.000	0.011	0.309	0.000	0.000	0.000	0.775		

**Table 3:** Estimates, corresponding standard errors in parentheses and p-values.

Innovation	Other parameters	Results
ZI-NB	$\hat{\nu }$	0.903
		(0.000)
		0.000
ZI-CMP	$\hat{\nu }$	1.058
		(0.001)
		0.000
ZI-PT	$\hat{\sigma^{2}}$	0.076
		(0.004)
		0.000
	$\hat{a}$	1.582
		(0.001)
		0.000
ZI-WCG	$\hat{\theta }$	0.223
		(0.000)
		0.000

The results in Tables 2 and 3 and Table 6 in S1 Appendix, were obtained assuming the training dataset from 18 March 2020 to 25 April 2021. It can be deduced that the Zero-Inflated Negative Binomial model (ZI-NB), given its lowest Akaike Information Criteria (AIC), outperformed the other competing ZI-PT, ZI-WCG and Poisson mixture models (See the results of Poisson mixture models in the S1 Appendix).

Referring to results in Tables 2 and 3, the variables ‘ReR’, ‘SI’, and ‘Vaccine’ were highly significant in reducing the number of infection in Mauritius, as compared to ‘CRW’.

The ReR is directly associated with the number of new active cases [57]. This is because by observing the evolution of the series, it can be deduced that in October 2020 when there were an increase in international mobility [19] following opening of frontier and in March 2021 when the second wave has resurfaced, an exponential increase in the number of active COVID-19 cases was reported. At this point, a worrisome ‘ReR’ of above 1 was being reported, indicating high risk of getting infected. Fortunately, based on these trends in ‘ReR’ and new COVID-19 active cases, the authorities triggered timely health related measures like vaccination campaigns in Mauritius and consequently, the policies proved its effectiveness in April 2021, with a reduction in the number of new active COVID-19 cases, and in the ‘ReR’. At this point, ‘ReR’ was below 0.5.

The event of vaccination indeed is playing a vital role in curbing the number in infection. Based on the reversed estimates of ‘Vaccines’, it can be deduced that as the vaccination campaigns take place, this is reflected positively in the share of Mauritian population which has already received at least one dose of the vaccines and likewise, the risk of getting infected is expected to decrease considerably. It has largely been proven that the COVID-19 vaccines reduces the overall attack rate by rendering the human immunity system more resilient. The chance for symptomatic and asymptomatic infections [58–63] and the severity of the symptoms [64, 65] are considerably reduced, thus entailing an adverse effect on the mortality rate related to COVID-19. More elaborated comments are provided below. Timely imposition of new immediate sanitary measures during the peak COVID-19 phases also play an important role in curbing the spread of the virus. In fact, the quicker and earlier the sanitary measures are imposed, the more rapidly is the SARS-CoV-2 contained in the local community. Conversely, unlike other European regions, Mauritius reported its highest cases of COVID-19 in both warmer and colder regions, and in both weather conditions—summer and Winter, so ‘CRW’ was proven to be insignificant in curbing the number of active COVID-19 cases. In fact, given the constant mutation of the SARS-CoV-2 in different regions and Mauritius having a comparatively restricted regional disparity, possibly a larger dataset on ‘CRW’ will allow better exploration of its association with the number of infection [66, 67].

Table 3 confirms that the estimates of the over-dispersion parameters in the ZI models are significant. In addition, the death series has also been analysed using the ZI-NB model due to its lower AIC. Below, the results have been presented.

From above Table 4, using the death series from 18 March 2020 till 25 April 2021, it can be concluded that all covariates except ‘CRW’ is highly significant in reducing the number of deaths related to COVID-19. The most important point to note is that in line with the results in Table 2 and as discussed in [58], the event of vaccination and the COVID-19 Stringency index have a substantial impact on the mortality rate related to COVID-19. As a matter of fact, in March and April 2021, Mauritius registered worrisome 8 deaths but once the vaccine coverage has widened and adherence to non-pharmaceutical interventions has increased, the number of death cases has dropped to zero.

**Table 4:** Estimates, corresponding standard errors in parentheses and p-values for death series under ZI-NB.

Intercept	ReR	SI	Vaccine	CRW	$\hat{\eta_{0}}$	$\hat{\eta_{1}}$	$\hat{\eta_{2}}$	$\hat{\eta_{3}}$	$\hat{\eta_{4}}$	AIC
0.537	0.001	-0.357	-0.625	-0.042	0.160	0.023	0.164	-0.027	0.165	10957.07
(0.000)	(0.000)	(0.000)	(0.000)	(0.101)	(0.010)	(0.001)	(0.001)	(0.001)	(0.016)	
0.000	0.000	0.000	0.000	0.678	0.000	0.000	0.000	0.000	0.000	

**Fig 5:**
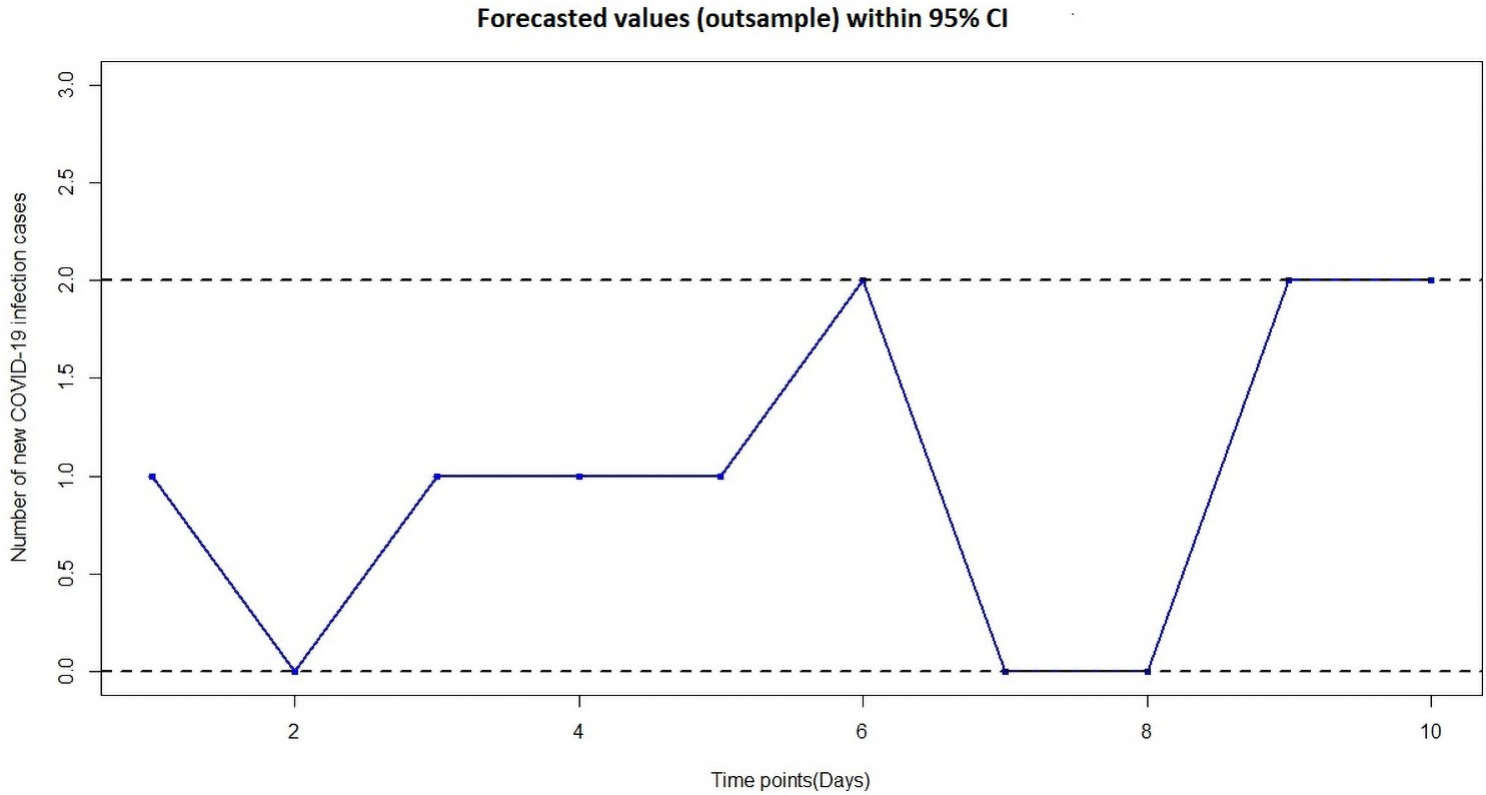
Forecasted values (out-sample) with 95% confidence interval.

**Fig 6:**
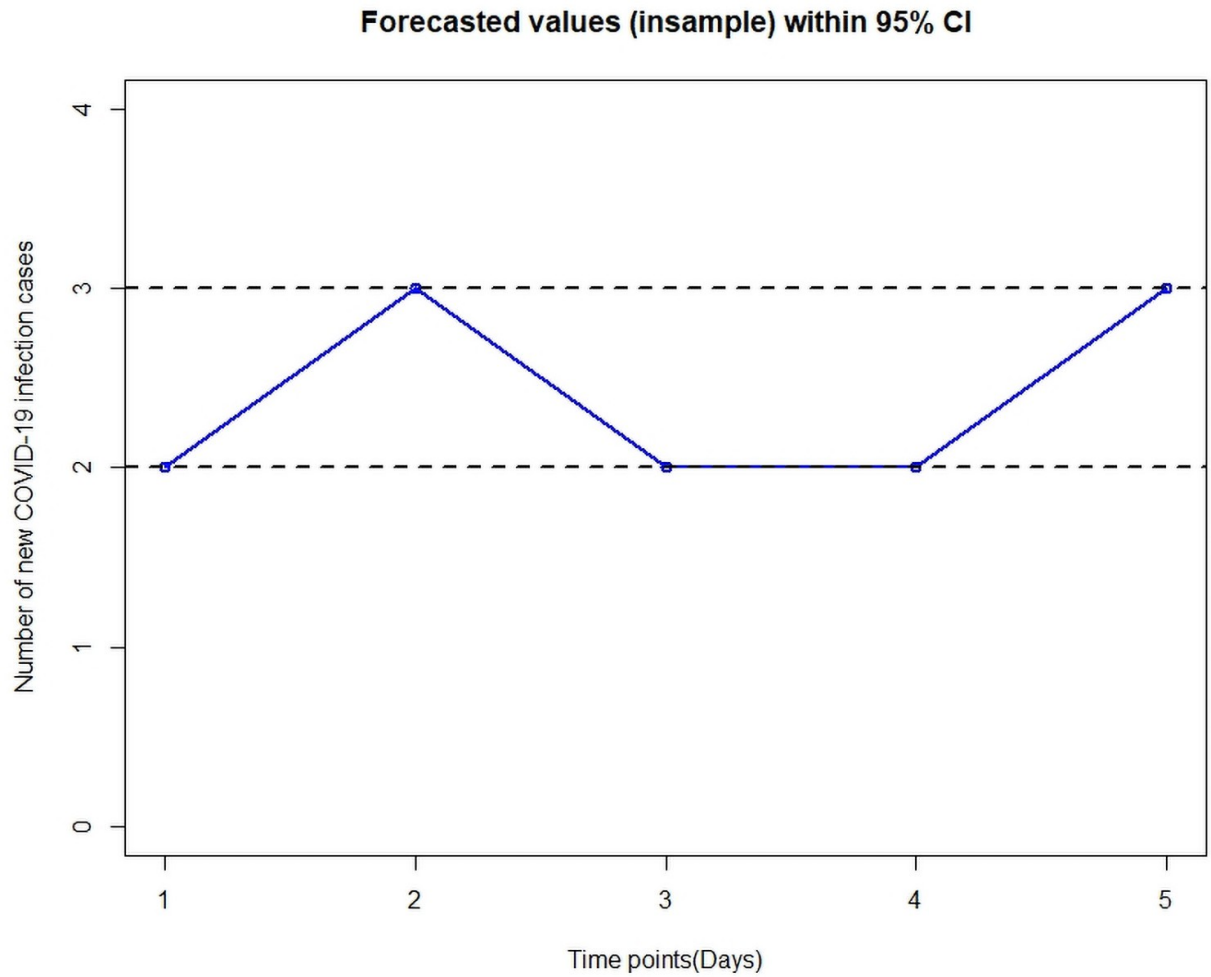
Forecasted values (in-sample) with 95% confidence interval.

Finally, we used the regression estimates for ZI-NB, as in Table 2, to conduct short term outsample forecasting of the number of new infected COVID-19 cases in Mauritius, from 26 April 2021 to 05 May 2021 and consequently, the ZI-NB model had the relatively lower Root Mean Square Errors (RMSEs) of 1.41. It can also be seen that the 95% confidence interval lies between 0 and 2 which means that during the next 10 days, that is from 26 April 2021 till 05 May 2021, there was 95% chance that the new COVID-19 infection case will lie between 0 and 2. In Fig 5 below, we demonstrate the 95% confidence interval plot:

An in-sample forecast for next 5 days, from 21 to 25 April 2021, with 95% confidence interval, showed that with a RMSE of 4.36, the ZI-NB model is relatively the better model. Below, in Fig 6, the CI plot has been illustrated:

Note that attention is drawn to the fact that due to some unmeasurable and unpredictable latent effects, the forecasted number of locally acquired new COVID-19 cases may not be easily estimated, especially in the long-run. In fact, in the rise of a sudden shock or spike, the forecasted values are naturally disrupted, since the predictor functions in the innovation distribution may not include a new physical or latent effect. Such a situation may be circumvented by updating the list of covariates on a daily basis and also by allowing the forecasts on a changepoint basis. Simultaneously, it is important to check the Variance Inflating factor (VIF) of the different regressors to avoid any multi-collinearity. Likewise, in the above analysis, the factor time was omitted due to the high VIF. We also note that in the high-ordered INAR process, the specification of latent effects may not be easily handled due to integrating the random effects (Refer to [1]).

## Discussion

In this study, useful INAR-type models were applied to the daily new COVID-19 infection cases and death cases while considering several covariates in order to understand the significant causes of the COVID-19 series and also to provide some reliable short term forecasts. Based on the above results, the event of Vaccination—’Vaccine’ and the COVID-19 Stringency Index—’SI’, are found to be highly significant in mitigating the spread of SARS-CoV-2 in the local Mauritian context and hence, the authorities can further work on strategies to re-enforce the ‘Vaccine’ and ‘SI’ measures. As part of COVID-19 preparedness plan, new and re-enforced COVID-19 legislations like the most recent “Restriction of Access to Specified Institutions”, upgrade in medical supplies and health equipment in terms of more personal protective equipment (PPE), high-tech protective masks like the novel ViriMASK, hospital beds for COVID-19 specialised hospitals amongst others, dynamic contact tracing teams, and more well-equipped laboratories for COVID-19 testing exercises, are further encouraged. Mauritius has it all but without the contributory support of the Mauritian population, nothing is worth. For a “COVID-19 free” Mauritius, concerned authorities are expected to boost the sensitization campaigns during this second wave of COVID-19 pandemic. Actually, slogans like “Sel Solution Vaccination” (Only solution is vaccination) are circulating on the social media but maybe by considering new motivating slogans like “Vacciner pu sauve nu pays” (Get vaccinated to save our motherland) can sensitize the population on the need to get vaccinated and the urgency to revive the Mauritian economy during this glooming economic scenario. Finally, the proposed INAR models can ultimately serve as an additional toolkit to the local authorities for better analysing and monitoring the evolution of the SARs-CoV-2 series in Mauritius.

## Supporting information

S1 Appendix (PDF)
