# Supplementary material for: Re-analyzing the SARS-CoV-2 series using an extended integer-valued time series models: A situational assessment of the COVID-19 in Mauritius
Source: PLoS One. 2022 Feb 8;17(2):e0263515. doi: 10.1371/journal.pone.0263515 (PMC8824322; doi:10.1371/journal.pone.0263515)
Supplement: S1 Appendix — (PDF) [file pone.0263515.s001.pdf]

Appendix

Test for Presence of trend

library(aTSA)
trend.test(covid$new_cases, method = c("cox.stuart"))
Approximate Cox-Stuart trend test
data: covid$new_cases
D- = 79, p-value = 4.818e-05
alternative hypothesis: data have a decreasing trend
trend.test(covid$deaths, method = c("cox.stuart"))
Approximate Cox-Stuart trend test
data: covid$deaths
D+ = 8, p-value = 0.06583
alternative hypothesis: data have a increasing trend

Test for Zero-Inflation - Vuong test

library(pscl)
glmpoisson = glm(abs(covid)~.,data=covidata, family="poisson");
zipoisson = zeroinfl(abs(covid)~.| 1, data = covidata,dist="poisson");
vuong(glmpoisson,zipoisson)
Vuong Non-Nested Hypothesis Test-Statistic:
(test-statistic is asymptotically distributed N(0,1) under the null that the models are indistinguishable)

	**Vuong z-statistic**	**H_A**	**p-value**
**Raw**	-7.116014 model2 >	model1	5.5546e-13
**AIC-corrected**	-7.105971 model2 >	model1	5.9740e-13
**BIC-corrected**	-7.085879 model2 >	model1	6.9082e-13

Table 5. Vuong test results

Poisson Mixture Results

Table 6. Estimates, corresponding standard errors in parentheses and p-values.

Innovation	$\hat{\rho_1}$	$\hat{\rho_2}$	$\hat{\rho_3}$	$\hat{\rho_4}$	$\hat{\rho_5}$	$\hat{\rho_6}$	$\hat{\rho_7}$	Intercept	ReR	SI	Vaccine	CRW	$\hat{\nu}$	$\hat{\theta}$	$\hat{\sigma^2}$	$\hat{a}$	AIC
NB	0.164	0.093	0.095	0.047	0.039	0.039	0.041	0.324	0.046	-0.182	-0.150	0.197	1.036				2166.0
	(0.180)	(0.115)	(0.024)	(1.217)	(0.115)	(0.756)	(0.599)	(0.001)	(0.001)	(0.001)	(0.004)	(0.005)	(0.002)				
	0.362	0.418	0.000	0.969	0.732	0.959	0.945	0.000	0.000	0.000	0.000	0.000	0.000				
Poisson	0.190	0.085	0.096	0.051	0.034	0.039	0.032	0.606	0.009	-0.398	-0.350	0.243					9555.9
	(0.010)	(0.011)	(0.007)	(0.007)	(0.001)	(0.014)	(0.010)	(0.010)	(0.003)	(0.000)	(0.010)	(0.010)					
	0.000	0.000	0.000	0.000	0.000	0.006	0.002	0.000	0.001	0.000	0.000	0.000					
CMP	0.031	0.015	0.073	0.054	0.020	0.049	0.037	-2.411	0.243	-1.189	-0.572	1.190	1.295				7673.8
	(0.567)	(0.797)	(0.325)	(0.086)	(0.771)	(0.366)	(0.572)	(0.154)	(0.006)	(0.006)	(0.154)	(0.337)	(0.010)				
	0.957	0.985	0.823	0.534	0.980	0.893	0.948	0.000	0.000	0.000	0.000	0.000	0.000				
WCG	0.196	0.103	0.104	0.052	0.042	0.040	0.031	0.206	0.114	-0.287	-0.319	0.112		0.109			8351.8
	(0.044)	(0.005)	(0.023)	(0.224)	(0.038)	(0.304)	(0.229)	(0.001)	(0.002)	(0.000)	(0.003)	(0.080)		(0.000)			
	0.000	0.000	0.000	0.817	0.279	0.894	0.892	0.000	0.000	0.000	0.000	0.158		0.000			
PT	0.018	0.013	0.013	0.061	0.040	0.037	0.040	0.301	0.053	-0.012	-0.181	0.082			0.081	1.096	8755.5
	(0.549)	(0.802)	(1.073)	(0.383)	(0.402)	(0.400)	(0.692)	(0.569)	(0.021)	(0.072)	(0.018)	(0.309)			(0.182)	(0.015)	
	0.974	0.987	0.990	0.874	0.921	0.927	0.954	0.597	0.011	0.006	0.000	0.792			0.656	0.000	
